# Leaf Senescence: The Chloroplast Connection Comes of Age

**DOI:** 10.3390/plants8110495

**Published:** 2019-11-12

**Authors:** Martín L. Mayta, Mohammad-Reza Hajirezaei, Néstor Carrillo, Anabella F. Lodeyro

**Affiliations:** 1 Instituto de Biología Molecular y Celular de Rosario (IBR-UNR/CONICET), Facultad de Ciencias Bioquímicas y Farmacéuticas, Universidad Nacional de Rosario (UNR), 2000 Rosario, Argentina; mayta@ibr-conicet.gov.ar; 2 Leibniz Institute of Plant Genetics and Crop Plant Research, OT Gatersleben, Corrensstrasse, D-06466 Stadt Seeland, Germany; mohammad@ipk-gatersleben.de

**Keywords:** senescence, reactive oxygen species, chloroplast, phytohormones, photosynthetic electron transport chain

## Abstract

Leaf senescence is a developmental process critical for plant fitness, which involves genetically controlled cell death and ordered disassembly of macromolecules for reallocating nutrients to juvenile and reproductive organs. While natural leaf senescence is primarily associated with aging, it can also be induced by environmental and nutritional inputs including biotic and abiotic stresses, darkness, phytohormones and oxidants. Reactive oxygen species (ROS) are a common thread in stress-dependent cell death and also increase during leaf senescence. Involvement of chloroplast redox chemistry (including ROS propagation) in modulating cell death is well supported, with photosynthesis playing a crucial role in providing redox-based signals to this process. While chloroplast contribution to senescence received less attention, recent findings indicate that changes in the redox poise of these organelles strongly affect senescence timing and progress. In this review, the involvement of chloroplasts in leaf senescence execution is critically assessed in relation to available evidence and the role played by environmental and developmental cues such as stress and phytohormones. The collected results indicate that chloroplasts could cooperate with other redox sources (e.g., mitochondria) and signaling molecules to initiate the committed steps of leaf senescence for a best use of the recycled nutrients in plant reproduction.

## 1. Introduction

Leaf senescence is an ordered physiological process in which cellular structures and biomolecules are progressively broken down and the resulting products mobilized to other plant organs such as fruits, seeds, tubers and/or more apical leaves [1,2,3]. The most visible manifestation of leaf senescence is yellowing caused by destruction of the chloroplast pigment-protein complexes and conversion of the constituent chlorophylls (*Chl*) into catabolic non-green derivatives after opening of the chlorin ring system [4,5]. Natural leaf senescence as it occurs in the field is normally age-dependent and accelerates upon transition of the vegetative into the reproductive growth phase [6,7].

Senescence is controlled by a genetic program involving major changes in expression patterns that result in degradation of cells targeted for demise and reallocation of the resulting products to the newly developing organs. Many genes induced during senescence (SAGs, for senescence-associated genes) encode enzymes involved in protein degradation, underscoring the relevance of nitrogen recycling during this process [8,9,10]. Mutants exhibiting delayed leaf senescence have been described in many species and are extremely useful to identify gene products involved in cell aging, cell death and nutrient salvage [1,11,12,13,14]. While all these mutants preserve leaf greenness for extended periods, it is convenient to distinguish functional mutants, in which the delay in senescence is coupled to preservation of metabolic capacity, from those that retain green color but show normal aging behavior [15]. The latter are just defective in *Chl* breakdown and were categorized as non-functional stay-green mutants, also termed cosmetic [16,17].

**Figure 1:**
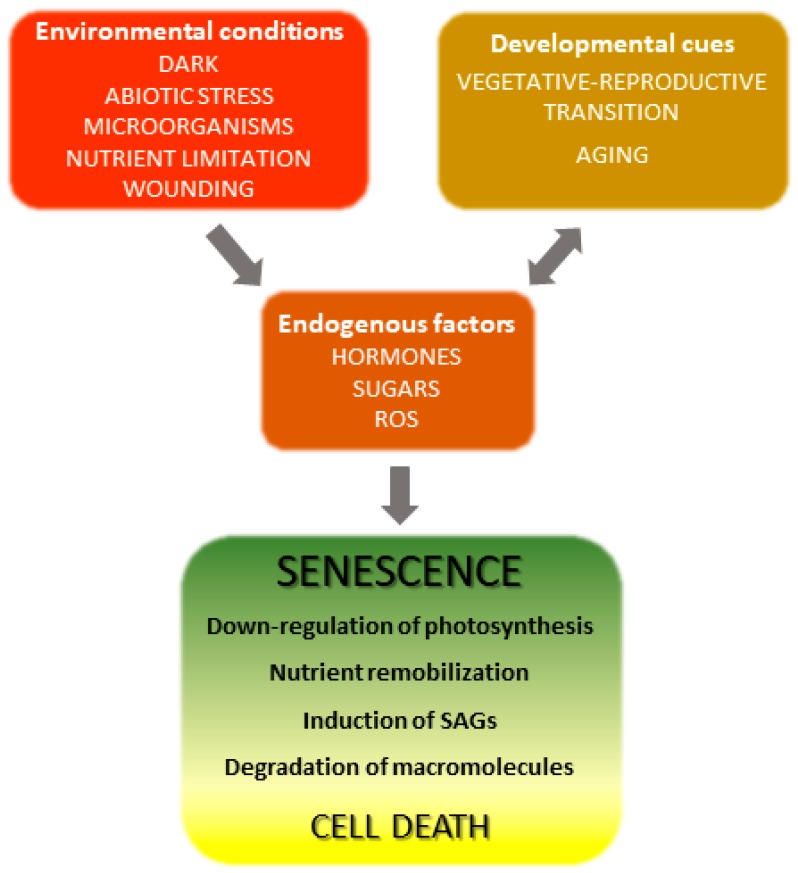
Overview of leaf senescence. The final stages of leaf development are basically determined by plant age and transition into the reproductive stage, but are also modulated by endogenous and exogenous cues which integrate into the developmental program. Environmental conditions affecting senescence progression include biotic and abiotic stresses and nutritional status, whereas hormones represent the most relevant endogenous factors. Many environmental stresses increase propagation of reactive oxygen species (ROS) in leaf tissue, which act as signaling molecules. SAGs, senescence-associated genes.

The onset and progression of the senescence process respond to developmental cues but are also affected by environmental factors (Figure 1) [18,19]. Indeed, senescence can be induced in otherwise young leaves by darkness, abiotic stresses and microorganisms [20,21,22,23,24]. Endogenous signaling molecules and pathways, including phytohormones, reactive oxygen species (ROS) and other redox-based signals, mediate the plant responses to these inputs, which in turn lead to extensive genetic, physiological and metabolic reprogramming.

**Figure 2:**
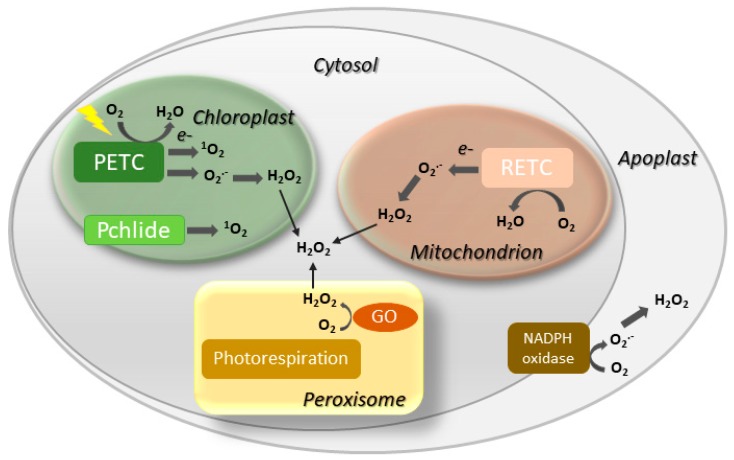
Major sites of ROS generation in the plant cell. GO, glycolate oxidase; Pchlide, protochlorophyllide; PETC, photosynthetic electron transport chain; RETC, respiratory electron transport chain. Disproportionation of O_2_^.−^ into H_2_O_2_ may be spontaneous or mediated by a suite of superoxide dismutases.

Involvement of ROS such as hydrogen peroxide (H_2_O_2_), singlet oxygen (^1^O_2_) and the superoxide (O_2_^.−^) radical in both natural and induced plant senescence is supported by many observations [25,26,27,28]. ROS can be produced in various cellular compartments through the activity of oxidases or as byproducts of oxido-reductive processes (Figure 2), and the contributions of the different sources to plant senescence are still poorly understood. In animal systems, ROS associated to mitochondrial metabolism play a central role in cell aging [29]. While a similar mechanism is likely to operate in nonphotosynthetic tissues [30,31], chloroplasts are the main ROS-producing organelle in illuminated leaves, whereas peroxisomes make a substantial contribution under photorespiratory conditions in C3 plants [32,33].

Although our knowledge on the participation of chloroplast redox chemistry in plant senescence and programmed cell death lags behind that of animal mitochondria, an increasing number of studies indicate that plastids might be playing a more important role than thought before during leaf senescence. The aim of this article is to critically review the evidence that supports this connection and to identify future research trends in the area. Since leaf senescence is not only a very interesting and significant biological question [3,34], but also bears relevance for agriculture [1,35,36], understanding the molecular mechanisms that underlie this developmental process might open new avenues to increase crop yield through an extended provision of leaf photosynthates to fruits, seeds and tubers [6].

## 2. Leaf Senescence Is Modulated by Multiple Inputs

**Figure 3:**
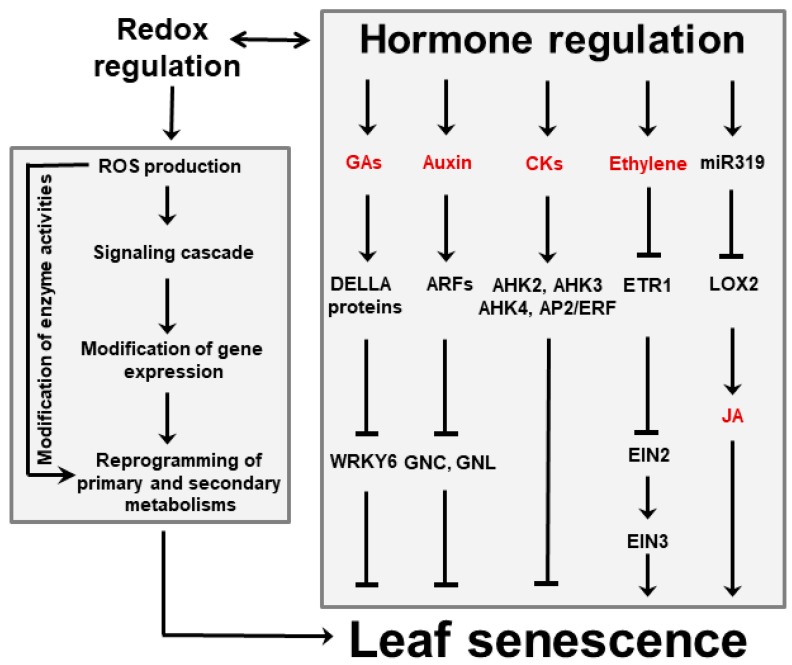
Interactions between hormones and ROS during leaf senescence regulation. Plant hormones can affect leaf aging either directly or via signaling cascades in which ROS are involved. Among them, GAs, auxins and CKs delay senescence, whereas ethylene and JA favor it. Regulation of JA accumulation via miR319 and lipoxygenase 2 (LOX2) is indicated. ROS might also act as independent signaling molecules that are produced upon leaf aging and stress conditions. ROS trigger modifications of gene expression that in turn result in reprogramming primary and secondary metabolisms involving sugars, energy, amino acids and antioxidants. Examples of reported interactions between hormones and ROS are given in the text. Abbreviations for hormones are also found there. AHK, Arabidopsis histidine kinase; AP2, Apetala 2; CKs, cytokinins; EIN, ethylene-insensitive protein; ERF, ethylene-responsive factor; ETR1, ethylene receptor 1; GNC, carbon-metabolism involved; GNL, GNC-like.

Several phytohormones influence leaf aging and cell death. Gibberellic acid (GA), auxins and cytokinins have been shown to delay senescence, whereas ethylene, jasmonic acid (JA), abscisic acid (ABA) and salicylic acid (SA) accelerate it (Figure 3) [18,37]. Cytokinins are able to retard senescence in plants and detached leaves, preventing *Chl* degradation and destruction of metabolic activity [38,39]. Conversely, a decline of the cytokinin pool is often accompanied by a decrease of photosynthetic activity and enhanced senescence. Auxins play a similar role by modulating expression of several auxin-responsive transcription factors (ARFs, Figure 3), which affect several processes associated with leaf senescence [40]. Moreover, increased expression of YUC6, a gene encoding a flavin-containing monooxygenase that catalyzes the rate-limiting step of auxin biosynthesis, was shown to delay senescence in transgenic Arabidopsis plants [41]. A different class of anti-senescence phytohormones is represented by pentacyclic diterpenes of the GA family, which act in a defined time-frame as the active GA forms are progressively degraded with leaf aging [42]. Treatment with inhibitors of GA synthesis led to increased ABA contents and promotion of senescence, suggesting that GA and ABA have antagonistic functions during this process [43].

Besides ABA, ethylene and JA are emerging as key players in senescence induction. They act directly or via interactions with each other, and involve various transcription factors and microRNAs (miR319, Figure 3). The relationship between ethylene and senescence has been best studied using Arabidopsis ethylene-insensitive mutants which display delayed leaf senescence [44]. Likewise, studies using Arabidopsis mutants exhibiting reduced JA levels or insensitivity to JA signaling revealed that the onset of natural and dark-induced senescence was delayed as the levels or sensitivity to this hormone declined [45,46].

ROS, phytohormones and environmental inputs do not operate through independent pathways, but instead display a significant degree of interaction and cross-talk. For instance, Merewitz et al. [47,48] have shown that higher cytokinin contents, obtained by over-expression of rate-limiting biosynthetic enzymes, resulted in induction of stress-responsive proteins such as antioxidants and chaperones, and led to improved drought tolerance. Conversely, ethylene biosynthesis during age-dependent and dark-induced leaf senescence were mediated by phytochrome-interacting transcription factors in Arabidopsis [49]. Interactions between ROS and phytohormones on the regulation of the senescence process have been demonstrated for auxins [40], GA [50,51] and SA [52]. Finally, many plant developmental programs depend on the combined action of several hormones that interact cooperatively or antagonistically. Cytokinin, in particular, has been shown to participate in regulatory networks with auxins, GA, ABA and strigolactones [53], and cross-talk between cytokinin and ethylene signaling pathways regulates leaf abscission in cotton during chemically induced senescence [54].

## 3. Senescence and Cell Death

The ultimate outcome of senescence is cell death and the two terms are sometimes employed indistinctly, but death is only the final stage of a complex and ordered physiological process. Indeed, there is a clear distinction between the tightly regulated senescence program that requires living cells and nutrient recycling, and the irreversible terminal phase of cell death, despite the common players and pathways they might share [7]. Similar to senescence, cell death can be induced independently of tissue age by many environmental stimuli including abiotic stress, pathogens, nutritional shortage and xenobiotics [1,55]. Biotic interactions offer a particularly rich set of examples. Biotrophic pathogens require living tissue to grow and manipulate host physiology to obtain nutrients, whereas necrotrophs kill host cells and feed on them [56,57,58]. Plants exposed to invading microorganisms often elicit a multigenic response termed the hypersensitive reaction (HR) that leads in most cases to localized cell death (LCD) at the site of infection [56]. It is assumed that the LCD associated to the HR helps to contain biotrophs by opposing a barrier of dead cells which deter their advance into the adjacent living tissue [56]. Infection by necrotrophic pathogens, instead, is facilitated by cell death and some necrotrophs may even promote host LCD in their own benefit [57].

As in the case of senescence, increased ROS production is a quasi-universal feature of cell death induced by environmental stresses and the HR [59,60,61,62,63]. In leaves, a significant fraction of all ROS are generated as byproducts of photosynthetic electron transport (Figure 2). Partial reduction of oxygen at the level of photosystem (PS) I and PSII renders O_2_^.−^ and, after spontaneous or enzyme-mediated disproportionation, H_2_O_2_ [64,65]. Singlet oxygen is usually produced in PSII by energy transfer from excited triplet-state *Chl* moieties to basal triplet-state oxygen resulting in spin inversion and several-fold increase in reactivity [66]. Under normal photosynthetic conditions, ROS build-up is limited by the action of antioxidant enzymes and reductants, but whenever distribution of reducing equivalents is perturbed by biotic or abiotic stresses, the rate of leakage increases dramatically, overcoming the control devices and leading to ROS propagation [67,68].

Another major source of chloroplast ROS is *Chl* metabolism (Figure 2). Many biosynthetic intermediates are loosely bound to the thylakoids without associating with reaction centers or antenna [69]. They are unable to participate in photosynthesis but can still be excited to their triplet state in the light, readily reacting with oxygen and propagating ^1^O_2_. *Chl* breakdown products released from the photosystems can also engage in this energy transfer reaction [5], and have been proposed to promote cell death during pathogen-induced HR [70].

The first report linking chloroplast redox processes to cell death came from the research of Samuilov et al. [71], who observed that cyanide treatment caused light-dependent destruction of chloroplast-containing leaf guard cells, whereas heterotrophic epidermal tissue was not affected. Photosynthesis-associated events such as over-reduction of plastoquinone and ROS build-up were proposed to mediate this effect [71]. Heat-induced cell death has also been linked to chloroplast redox chemistry [72,73]. A complete death program is triggered by ^1^O_2_ via the plastidic proteins Executioner 1 and 2, as revealed by experiments on the Arabidopsis *flu* mutants, which accumulate the photosensitive biosynthetic intermediate protochlorophyllide in the dark and propagates ^1^O_2_ upon illumination (Figure 2; [74]). Moreover, cell death associated to the HR is significantly reduced in the absence of light [75,76], and can be abolished by targeting antioxidant proteins to chloroplasts [77]. In addition to these examples, many other reports identified chloroplast ROS as key players in the signaling events that lead to programmed cell death [69,78,79,80]. In contrast, little is known on the nature of the death-promoting signals that exit the chloroplast, and the relationship of the cell death executioners with the organelle.

## 4. Senescence and Chloroplasts

Results discussed in the preceding sections illustrate that the relationship between chloroplast ROS propagation and stress-associated cell death is amply supported by experimental data. By contrast, the empirical evidence linking plastid redox processes with natural senescence is scant. It should be borne in mind, within this context, that while all plant organs senesce at the end of their life cycles, most of them do not have chloroplasts, indicating that different types of signaling networks could be invoked in the various plant tissues. Despite this basic difference, ROS bursts have been shown to precede and accompany natural senescence of leaves, petals and fruits ([31], and references therein).

Initial evidence indicating that chloroplast-generated ROS could signal leaf senescence was provided by Zapata et al. [81], who reported that tobacco *ndhF* mutants exhibited delayed senescence. The plastome-located *ndhF* gene encodes a subunit of the NAD(P)H dehydrogenase-like complex (NDH) of chloroplasts and its mutation resulted in loss of activity. The dehydrogenase is proposed to mediate one of the cyclic electron transport (CET) routes existing in chloroplasts [82,83]. The authors proposed that reducing equivalents delivered by NDH to the photosynthetic electron transport chain (PETC) could be misrouted to oxygen in senescing leaves, leading to ROS production as it occurs with Complex I in mitochondria [84]. NDH inactivation in the mutant line would prevent this effect and generate a stay-green phenotype [81]. However, it is also likely that in the absence of NDH-dependent CET, other electron transfer pathways are favored as safety valves for the excess of reducing power, as illustrated by Krieger-Liszkay et al. [85]. Putative candidates might include lineal electron flow that would ultimately deliver electron to the Calvin–Benson cycle, the PROTON GRADIENT REGULATION 1-PGR5-like Photosynthetic Phenotype 1 (PGR5-PGRL1)-dependent CET pathway where electrons from ferredoxin (Fd) are cycled around PSI into the plastoquinone pool, and the plastid terminal oxidase [85], all of which can generate alternative signals affecting senescence outcome.

Increased levels of antioxidants usually retard senescence, and *vice versa*. For instance, knock-down of the rate-limiting enzyme of the tocopherol biosynthetic pathway homogentisate phytyltransferase by RNA-directed silencing led to decreased tocopherol levels, ROS build-up and accelerated senescence in tobacco [86]. About half of the total cellular tocopherol contents are associated to the thylakoid membrane, suggesting that their decline could impose oxidative stress preferentially in chloroplasts that might explain the early senescence of the transformants. Noteworthy, the differential effect displayed by the transgenic plants was only evident after flowering [86].

Involvement of chloroplast ROS on senescence was also suggested by the phenotype of wheat lines expressing a chloroplast protein kinase that inactivates thylakoid-bound ascorbate peroxidase (tAPX) and exhibited anticipated leaf senescence [87]. Noteworthy, expression of tAPX, but not the stromal APX isoform, specifically regulates cold priming of ROS signaling in Arabidopsis [88]. Suppression of dehydroascorbate reductase, responsible for regenerating ascorbate from an oxidized state, led to a similar phenotype [89], and reduction of ascorbate levels in *vtc1* (*vitamin c1*) mutants enhanced senescence-associated gene expression [90,91]. Finally, transgenic Arabidopsis plants with decreased chloroplastic glutathione reductase 2 activity (GR2) exhibited early senescence phenotypes and increased levels of the senescence markers *SAG12* and *SAG13* [92].

The PETC, with its hazardous combination of high-energy redox intermediates and oxygen evolving systems, is a prominent site of ROS propagation. Moreover, degradation of individual photosynthetic components usually precedes *Chl* loss [85,93], aggravating the leakage of reducing equivalents to adventitious electron acceptors such as oxygen. The order of disassembly during leaf senescence varies between species or even cultivars [85], disrupting complete sections of the PETC. Besides being particularly rich in detoxification systems, chloroplasts harbor a number of alternative electron transport pathways [94]. They play secondary roles in young or mature leaves with an intact PETC, but become important during the senescence-associated collapse of the photosynthetic apparatus, dissipating the excess of energy and reducing power while keeping ATP synthesis required for nutrient recycling and export [73,85,95]. ROS generated at the PETC could act as retrograde signals for senescence progression, either directly or through changes in hormone status, as has been reported for the chloroplast synthesis of SA [96]. Alternative electron transports, in turn, might keep ROS at signaling levels and prevent their build-up to a degree that can cause premature cell death and compromise nutrient remobilization.

While selective elimination of photosynthetic components helped to confirm involvement of the PETC redox poise in the development of the senescence program, introduction of alternative electron carriers generating novel transport pathways could help to identify the molecular nature of the retrograde signals transmitted by the chloroplast. A recent example of this strategy is provided by tobacco transformants expressing a plastid-targeted flavodoxin (Fld) from cyanobacteria [97]. Flavodoxins are flavin-containing proteins which functionally replace Fd, the final acceptor of the PETC, in cyanobacteria and some algae [98]. They are induced under situations of environmental hardship as an adaptive response to the stress-dependent decrease of Fd levels [98]. Fld-encoding genes are absent from plant genomes [99], but when expressed in plant chloroplasts the flavoprotein can accept reducing equivalents from the PETC, bypassing the limitations imposed by Fd decline and diverting these electrons from oxygen to productive pathways of the plastid. In doing so, it specifically prevents chloroplast ROS build-up under adverse situations and keeps the PETC in a more oxidized state, closer to physiological conditions [57,77,98,100].

Fd levels are also down-regulated during natural leaf senescence [101], and Fld was shown to inhibit ROS accumulation during this developmental process. Senescence was significantly delayed in the Fld transformants, with differential preservation of *Chl*, carotenoids, protein contents, cell and chloroplast structures, membrane integrity and cell viability [97]. Fld also improved maintenance of pigment-protein complexes, central metabolism including photosynthesis and levels of bioactive cytokinins and auxins in aging leaves. Delayed induction of SAGs indicated that the entire genetic program of senescence was affected by expression of the flavoprotein [97]. Not unexpectedly, chloroplast Fld activity also prevented cell death associated to environmental stresses and pathogens [57,77,98,100].

In animal systems, where mitochondrial ROS make a key contribution, dissociation of cytochrome *c* from the inner mitochondrial membrane of death-targeted cells results in over-reduction of up-chain respiratory transporters and increased ROS propagation by adventitious electron transfer to O_2_ [102]. Fd decline could play a similar pro-oxidant role in senescing leaves by introducing an acceptor side limitation on the PETC that could be circumvented by Fld expression [97].

Chloroplasts may also contribute to senescence-dependent cell death by releasing cytochrome *f* from thylakoids [32,103,104], much as cytochrome *c* in animals and yeast. Surprisingly, cytochrome *f* is translocated to the cytosol at an early stage of the senescence process in detached rice leaves, interacting with components of the proteasome system and inducing caspase-like activity. Moreover, addition of purified cytochrome *f* to cell-free extracts caused protease activation and DNA laddering resembling apoptosis, and its over-expression triggered cell death in isolated protoplasts [32]. While many aspects of the underlying mechanism still need to be worked out [79], the potential role of cytochrome *f* in chloroplast ROS propagation and senescence development certainly deserves further studies.

Hormonal changes go along with the regulation of chloroplast ROS homeostasis during senescence. ROS induce several hormone-responsive transcription factors during leaf senescence, such as those belonging to the NAM, ATAF1/2, CUC2 (NAC) and WRKY domain-containing (WRKY) gene families [105,106]. Interestingly, Guo et al. [52] have recently demonstrated that the interaction between ROS and SA is dependent on WRKY75 that in turn promotes SA synthesis by inducing expression of the *SA-Induction-Deficient 2* (*SID2*) gene, and suppresses H_2_O_2_ metabolism by inhibiting transcription of the gene-encoding peroxisomal catalase 2.

Thus, the evidence collected over the last years suggests an important function of chloroplast redox metabolism along with ROS production and hormonal regulation in senescence initiation and progression. Depending on the leaf developmental stage, plant hormones might be the initial triggers of leaf senescence followed by ROS production and concomitant activation of antioxidant metabolism.

## 5. Degradation of Chloroplast Components in Senescing Leaves Provides Most of the Nitrogen Required for Reproductive Development

During leaf senescence, thylakoid, stromal and envelope components are degraded and their catabolic products (mostly amino acids and lipids) are mobilized to sink organs. Nitrogen required for fruit and seed development is largely provided by breakdown of *Chl* and resident proteins, most conspicuously Rubisco [107], leading to a massive nutrient turnover involving more than 10 billion tons of protein and pigments recycled per year from aging leaves ([108], and references therein). Rubisco alone represents about 50% of total leaf nitrogen in C3 plants [85].

*Chl* degradation includes catabolic steps in both plastids and vacuoles, and needs to be tightly controlled because, as indicated previously, intermediate products can cause oxidative damage under excess light or other forms of stress [5]. The initial reactions leading to accumulation of the primary fluorescent breakdown products take place in chloroplasts. These intermediates are subsequently exported to the cytoplasm and the vacuole, where they are converted into phyllobilins, the end-products of *Chl* degradation [109]. Carotenoids, on the other hand, are released from the photosynthetic protein complexes of senescing leaves and integrated into plastoglobules where they are degraded by committed dioxygenases [110].

As in the case of *Chl*, chloroplast proteases participate in the initial breakdown of Rubisco and other plastidic proteins [111]. More than twenty of these proteases have been identified so far, some of which are associated with senescence [112]. Aspartic protease CND41 cleaves Rubisco [113], whereas degradation of the thylakoid D1 protein is mediated by stroma-localized serine endopeptidase DEGP2 and metalloprotease FTSH [32]. At least one metacaspase was localized to plastids in Arabidopsis, and reported to be upregulated upon exposure to biotic and abiotic stresses [114].

While this initial plastid-dependent protein degradation certainly plays a role in nitrogen salvage, most of the proteolytic activity induced during leaf senescence localizes to small vacuoles that bud off from chloroplasts, first identified in aging leaves from Arabidopsis [115,116]. These vesicles are 0.8–1 μm in diameter and were defined as senescence-associated vacuoles (SAVs) based on the presence of a single membrane containing vacuolar H^+^-pyrophosphatase and a low pH lumen, although they are even more acidic than the central vacuole [8,108]. The cysteine protease encoded by the *SAG12* gene localized to SAVs, as revealed by activity measurements [117], and the inhibition of proteolytic activity in isolated SAVs by a cysteine protease inhibitor [115]. However, SAG12 is not the only protease present in these vacuoles, since SAVs isolated from Arabidopsis *SAG12* mutants still display significant protease activity [117].

A second extrachloroplastic degradation pathway involves vesicles containing CV, a plastid-targeted protein with a predicted transmembrane domain that interacts with endogenous DEGP and FTSH proteases [32]. CV-containing vesicles projecting from chloroplasts carry thylakoid, stromal, and envelope proteins, but apparently no plastoglobules, and are subsequently delivered to vacuoles for degradation. CV expression is activated during senescence and abiotic stresses. Overexpression of CV accelerated senescence and chloroplast dismantling, while knock-down of the CV-encoding gene increased chloroplast stability under abiotic stress conditions [32].

Nitrogen salvage pathways involving SAVs and CV-containing vesicles do not depend on autophagy, whereas other degradative routes, including formation and processing of Rubisco-containing bodies, are autophagy-dependent [8,108,118] and respond to photosynthetic sugar production [119]. Formation of these bodies, which are projected from chloroplasts as stromules and contain both the large and small subunits of Rubisco, was shown to be upregulated during natural and dark-induced senescence, suggesting that this pathway is important for nutrient remobilization [107,120]. It is worth noting, within this context, that ROS significantly increase the susceptibility of Rubisco to degradation by specific proteases [121].

## 6. Senescence in Nonphotosynthetic Plant Organs

Chloroplasts progressively differentiate into chromoplasts during petal development and fruit ripening [122,123], a transition that involves inactivation of photosynthesis, degradation of *Chl*-containing complexes and de novo synthesis of the carotenoids and xanthophylls that lend these organs their typical colors. The two processes, e.g., *Chl* breakdown and carotene synthesis, can be genetically uncoupled as in the *green flesh* mutant of tomato, which accumulates *Chl* and lycopene in the same plastid [123]. First visible symptoms of petal and fruit senescence are wilting and softening, respectively, often accompanied by color changes [30,124]. As most chloroplasts have disappeared from these organs long before the onset of senescence, the origin(s) of the ROS that normally accompany this process need to be different, and ROS-dependent pathways may diverge significantly from those operating in leaves [30,31].

Main sources of ROS in petals are likely to be mitochondria but also peroxisomes, as indicated by experiments with amidotriazole, which stimulates peroxisomal H_2_O_2_ production [125]. Nutrient remobilization from senescing petals is rather modest compared to leaves. Actually, petal senescence proceeds rapidly in many species, suggesting that quick elimination of these energy costly organs is the main goal of the program [126]. Cytokonins have an anti-senescence effect as in leaves [127], whereas ethylene favors the process [128].

Mitochondria were also reported to be main players during dark-induced leaf senescence [129]. The study of senescence in nonphotosynthetic organs is at a preliminary stage. Comprehensive reviews describing the most relevant findings and research trends in the field have been published recently [30,31], and readers are referred to them for a more detailed description of this very important aspect of plant physiology.

## 7. Redox Signaling and the Chloroplast Connection

A direct question arising from the correlation between chloroplast ROS build-up and leaf senescence reviewed in the preceding sections is whether this correspondence actually reflects a functional interaction. In other words, if the redox changes undergone by the plastid act as signals required for senescence itself. To answer this question, several aspects should be considered, most importantly the identity of the reactive species involved, the signaling routes and the potential targets of this modulation.

In discussing the possible roles played by ROS during senescence, it is important to consider that different ROS have distinct properties and stability [33]. Furthermore, they are expected to interact with and modify other signaling molecules, reactions that depend on their chemical properties and reactivity. The O_2_^.−^ radical displays a half-life of about 50 ms in living cells, resulting in a predicted diffusion distance of ~40 μm [130]. Preferred targets include [4S-4Fe] clusters of hydrolyases [131]. Hydrogen peroxide, with a half-life that can reach several minutes in biological media, is able to cross membranes by simple diffusion or through water-transport systems such as aquaporins. It reacts with protein thiols and double bonds as those present in unsaturated fatty acids, whose oxidation leads to the formation of organic ROS (mostly lipid peroxides) and self-propagating rounds of oxidative reactions [130,132]. Singlet oxygen has a half-life in the order of 1 μs, but its site of formation usually limits its potential targets to chlorophylls and membranes.

Genome-wide analysis of transcriptional profiles indicate that individual ROS (^1^O_2_, O_2_^.−^ and H_2_O_2_ have been assayed) elicit specific changes in gene expression with both common and idiosyncratic elements compared to the other reactive species [133,134,135,136]. Several ROS-responsive genes encode transcription factors, which in turn regulate the expression of a large number of effector genes [137,138,139]. The well-defined stages of Arabidopsis leaf senescence were exploited to investigate the expression of ROS-related genes along leaf aging [139,140]. Transcription factors belonging to the WRKY and NAC families were particularly enriched among differentially expressed genes. When transcript patterns were compared between senescing leaves and petals, two distinct sets of sequences were obtained for the two tissues [30,133]. These unique signatures presumably reflect the different origin of the signaling ROS involved, chloroplastic versus mitochondrial. Accordingly, out of 23 ROS-responsive transcription factors upregulated in Arabidopsis leaves during dark-induced senescence (when no chloroplast ROS are expected to accumulate), 21 showed similar behavior or no change in senescing petals [30,106], underscoring the role played by mitochondria during senescence in nonphotosynthetic organs or conditions.

**Figure 4:**
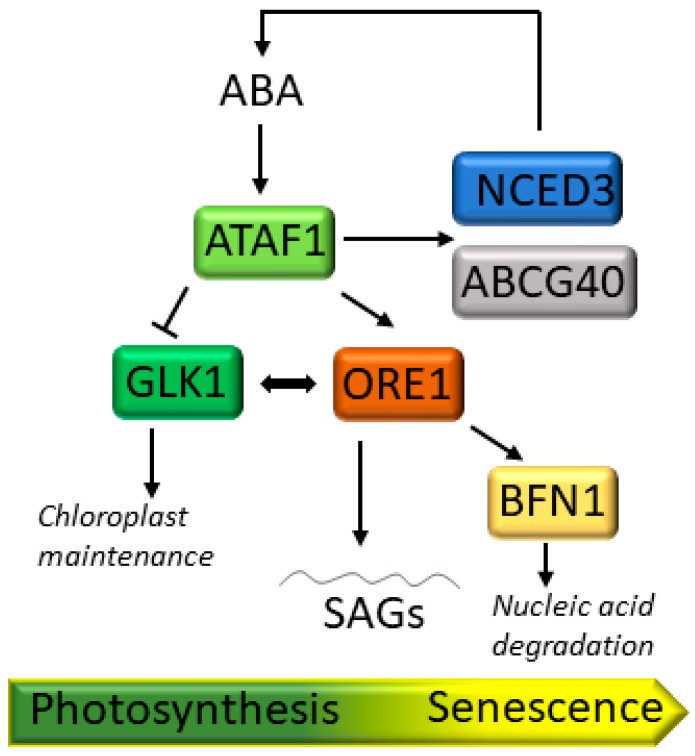
Schematic model highlighting ACTIVATING FACTOR1 (ATAF1) regulatory network during senescence. ATAF1 activates ORESARA1 (ORE1) and represses GOLDEN-LIKE1 (GLK1) expression by directly binding to the promoters of both genes. ORE1 directly interacts with GLK1, which is involved in the proper development and maintenance of chloroplasts. During senescence progression, the expression of GLK target genes is impaired, while the expression of ORE1 target genes is enhanced, promoting developmental senescence and programed cell death through a gene regulatory network that involves BIFUNCTIONAL NUCLEASE1 (BFN1) and other direct target genes, including different SAGs. BFN1 plays a role in nucleic acid degradation. Besides, ATAF1 also elevates the ABA levels by directly interacting with the ABA biosynthetic gene *NCED3*, encoding 9-*cis*-epoxycarotenoid dioxygenase 3, and the ABA transporter ABCG40.

Senescence is coupled to the inactivation of photosynthesis through the ABA- and H_2_O_2_-responsive transcription factor *ACTIVATING FACTOR1* (*ATAF1*) which induces expression of the transcriptional regulator ORESARA1 (ORE1) and represses the *GOLDEN-LIKE1* (*GLK1*) gene by directly binding to their promoters (Figure 4; [28]). ORE1 promotes senescence [141] whereas GLK1 is involved in chloroplast maintenance [142]. Downstream targets of *GLK1* include several genes encoding light-harvesting *Chl a/b*-binding proteins, indicating that its repression results in faulty assembly of pigment-protein complexes, inhibition of photosynthesis and increased ROS propagation in chloroplasts. Then, *ATAF1* activity shifts the cellular balance against growth and metabolism and toward chloroplast ROS build-up and the progression of senescence (Figure 4; [28]).

Analysis of pro-oxidant and antioxidant activities during leaf aging indicates a decrease in superoxide dismutase activity that converts O_2_^.−^ into H_2_O_2_, while the thylakoid NDH complex, which favors the generation of ^1^O_2_ and O_2_^.−^ [81], and peroxidase activities consuming H_2_O_2_, increased [143]. Then, the (^1^O_2_ + O_2_^.−^)/H_2_O_2_ ratio is expected to rise during senescence previous to cell death, and Sabater and Martín [143] postulated that changes in this ratio could determine the activation of the death network response of the cell. It should be noted, however, that ROS are able to create localized oxidative environments that might facilitate signaling by other pathways, such as calcium mobilization, thiol–disulfide exchange, protein–protein interactions, and differential binding and activation of transcription factors. How the balance between ROS production and elimination is regulated, and how signals are transmitted to the cell machinery to trigger senescence processes in plant organs are key to understand this very intriguing biological process.

## 8. Concluding Remarks

Chloroplasts are main targets for degradation during leaf senescence and provide the bulk of recycled nutrients for the development of reproductive organs, especially nitrogen compounds resulting from protein and *Chl* breakdown. Results obtained in the last few years, however, indicate that chloroplasts also contribute signals that affect leaf senescence at an early stage and at a hierarchically high level of developmental decisions [32,86,87,88,89,97]. The nature of these signals remains yet to be determined, but they could be linked to oxido-reductive pathways of the organelle, in particular to the redox status of the PETC.

In this sense, intervention of the PETC with alternative electron shuttles from plants or cyanobacteria (as exemplified by Fld) has shown promise to understand (and manipulate) the retrograde signals transmitted by the chloroplasts that delay or accelerate leaf senescence. Various electron transport pathways can be bolstered, attenuated or introduced *de novo* that exchange energy and reducing equivalents with different components of the PETC. They include, among others, NDH, plastid terminal oxidase, CET via the PGR5-PGRL1 complex, energy dissipation through nonphotochemical processes, and flavin-diiron proteins. Depending on the redox partner(s) introduced, the interventions will allow entire sections of the PETC to become more oxidized or over-reduced, suppress ROS generation or favor particular species against others, providing customized tools to probe the effects of these changes on leaf senescence. Application of this approach to model and crop plants will provide a richness of new data on the connection between chloroplast redox status and leaf aging.

Cell death as the final stage of leaf senescence shares many features with those caused by environmental stresses and pathogens. Identification of the common players involved in these processes is a most rewarding objective, not only due to the scientific relevance of the biological mechanisms involved but also to combine functional lifespan with stress endurance through minimal genetic interventions. Also in this case, manipulation of the PETC offers opportunities for improvement, as shown by Fld expression in tobacco plants which led to a stay-green phenotype, increased stress tolerance and suppression of cell death during the HR elicited by a non-virulent microorganism.

Leaf senescence is of critical importance for crop yield. Premature senescence, as that caused by environmental adversities, is known to negatively affect plant productivity. At the same time, nitrogen recycling requires that some organs senesce and die for others to develop. Then, optimization of senescence timing for defined species and growth conditions represents a major goal of crop breeding programs. In a recent review, Krieger-Liszkay et al. [85] provided insights into the role played by chloroplast ROS and alternative electron transports during senescence, and highlighted the relevance of maintaining ATP synthesis at advanced stages of this process. We stress herein the possibilities offered by manipulation of the PETC to generate crops with extended functional lifespans in the fluctuating conditions faced by plants growing in the field.
